# Determinants of Normal Left Atrial Volume in Heart Failure with Moderate-to-Severely Reduced Ejection Fraction

**DOI:** 10.1155/2018/7512758

**Published:** 2018-04-23

**Authors:** Dipika Gopal, Jing Wang, Yuchi Han

**Affiliations:** ^1^Cardiovascular Division, Department of Medicine, Perelman School of Medicine of the University of Pennsylvania, Philadelphia, PA 19104, USA; ^2^Department of Cardiology, PLA General Hospital, Beijing, China

## Abstract

**Background:**

Indexed left atrial volume (LAVi) is a robust predictor of adverse cardiovascular events. A minority of patients with moderate-to-severe left ventricular (LV) systolic dysfunction maintain normal LAVi. We followed clinical and echocardiographic parameters for at least 6 months to understand how this population is different from patients with similar systolic dysfunction and dilated left atria.

**Methods and Results:**

We searched our electronic medical records for “normal” (*n*=817) and “severely dilated” (*n*=1094) LA size and LV ejection fraction (EF) ≤ 35% on echocardiogram reports from 2009 to 2015. We analyzed 115 subjects for LAVi, biplane EF, and diastolic parameters over 2 echocardiograms at least 6 months apart. Younger age, white race, being on an angiotensin-converting enzyme inhibitor, smaller end-diastolic LV volume (LVEDV), and longer deceleration time (DT) were associated with having a normal LAVi. The receiver-operating characteristic curve has an area under the curve of 0.95 (*p* < 0.0001) for this model. An increase in LVESVi and early mitral flow velocity and a decrease in DT explain 32% of the variance seen in LAVi increase over time.

**Conclusion:**

In patients with moderate-to-severely reduced EF, younger age, being on heart failure therapies, and better diastolic dysfunction were independently associated with a normal LAVi. Improvement in systolic and diastolic performances was associated with decreasing LAVi with 6-month to 1-year follow-up.

## 1. Introduction

Indexed left atrial volume by body surface area (LAVi) is a robust predictor of advanced cardiovascular diseases, including atrial fibrillation, heart failure, stroke, and mortality [1–4]. In the absence of aortic or mitral valve disease, hypertension and left ventricular hypertrophy can lead to chronic progressive pressure and volume overload causing left atrial (LA) enlargement over time [1, 5, 6]. The LA size has emerged as an imaging marker of diastolic dysfunction in patients who have heart failure with preserved left ventricular (LV) ejection fraction (EF) [6–8]. Among patients with systolic heart failure with reduced LVEF, it is shown that enlarged LA size offers incremental prognostic values in the elderly population [9]. While several theories exist correlating increased LA size with progressive systolic and diastolic dysfunction, little is known about the minority of systolic heart failure patients whose LA size remains normal. This study aims to compare clinical factors and echocardiographic parameters longitudinally to further our understanding of what differentiates this small population from those with severely dilated LA size in the setting of reduced LVEF.

## 2. Methods

### 2.1. Subject Selection

**Figure 1:**
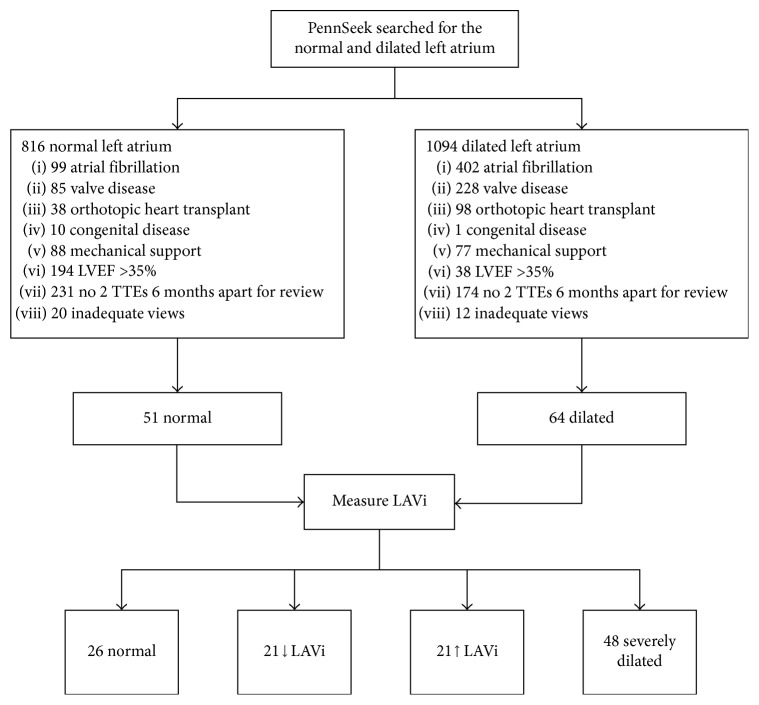
Patient selection. The above exclusion criteria were used to generate the final cohort of patients for analysis. Not shown are the 33 patients that had mild and moderately dilated LAVi after area-length measurement. LVEF, left ventricular ejection fraction; TTE, echocardiogram; LAVi, indexed left atrial volume.

We searched our electronic medical records using a natural language search tool PennSeek for “normal” and “severely dilated” LA size and “severely depressed ejection fraction” or “EF ≤ 35%” (visual estimate) on the echocardiogram (TTE) reports from 2009 to 2015. From this list, we excluded subjects on mechanical circulatory support, with heart transplant, greater than mild valvular heart disease, congenital heart disease, and atrial fibrillation. Only subjects with at least 2 TTEs 6 months apart with adequate images for assessment were included. Subgroup analysis was performed on a select group with at least 1 year between the baseline and follow-up TTE. We then performed biplane LVEF on these TTEs and excluded those with LVEF > 40% on the baseline TTE (Figure 1). Baseline demographics, past medical history, biomarkers, and medications were obtained from chart review. Medications including angiotensin-converting enzyme inhibitors (ACE-Is), angiotensin receptor blockers, aldosterone antagonists, nitrates, and hydralazine were recorded for both TTE time points, whereas the other medications were recorded at baseline only. Coronary artery disease was defined as greater than 70% stenosis in one or more coronary vessels on coronary angiogram within 3 months of the first TTE, evidence of coronary revascularization with either surgery or stenting, or positive stress test within 3 months of the first TTE. History of hypertension, hyperlipidemia, diabetes, stroke/transient ischemic attack, obstructive sleep apnea, smoking, and death data were obtained from the medical record. Our institutional review board approved this retrospective review study with waiver of consent.

### 2.2. Imaging Analysis

**Figure 2:**
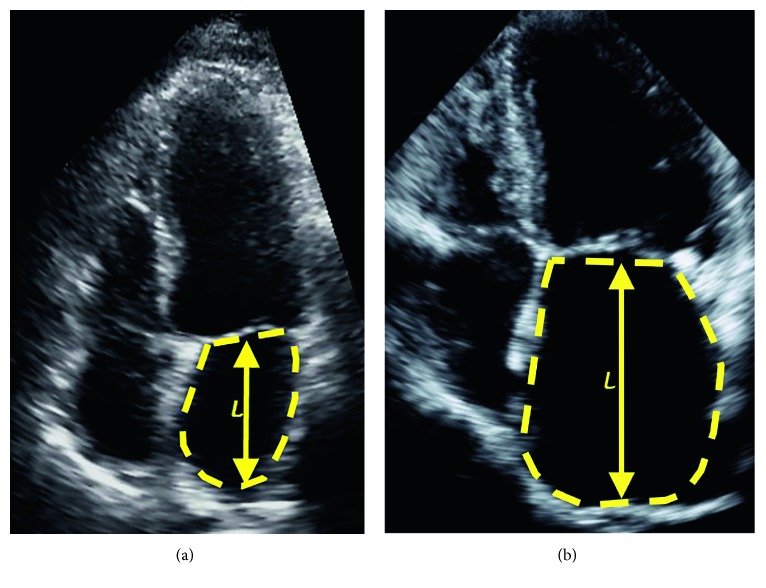
LAVi measurement. A 4-chamber area-length calculation of a normal (a) and dilated (b) atrium in mL/m^2^ indexed by body surface area. A 2-chamber component of calculation is not shown. LAVi, indexed left atrial volume.

We measured LAVi by the biplane area-length method using apical 2- and 4-chamber views and indexed by body surface area (Figure 2). Volume measurements were classified into persistently normal, mild and moderately dilated, and severely dilated by the 2015 American Society of Echocardiography guidelines (normal: 16–34 ml/m^2^, mild and moderate: 35–48 ml/m^2^, and severe: >48 ml/m^2^) [10]. Measurements were performed by two observers after both measured a sample population to ensure <10% interobserver variability. LVEF was calculated from apical 2- and 4-chamber views using the modified Simpson's method. Mitral inflow measurements (early and late diastolic velocities and deceleration time) were obtained from pulsed-wave Doppler in the apical 4-chamber view. Mitral annular velocities were measured from tissue Doppler images in the apical 4-chamber view. One independent experienced observer blinded to clinical data performed biplane LVEF and diastolic measurements.

### 2.3. Statistical Analysis

The baseline characteristics of the study population were summarized by normal LAVi versus dilated LAVi groups as defined above. Continuous variables were expressed as mean ± standard deviation and compared using a 2-group *t*-test. Categorical variables were expressed as count and frequency and were compared using a chi-squared test. The significance was considered at the 2-sided alpha level of 0.05.

**Figure 3:**
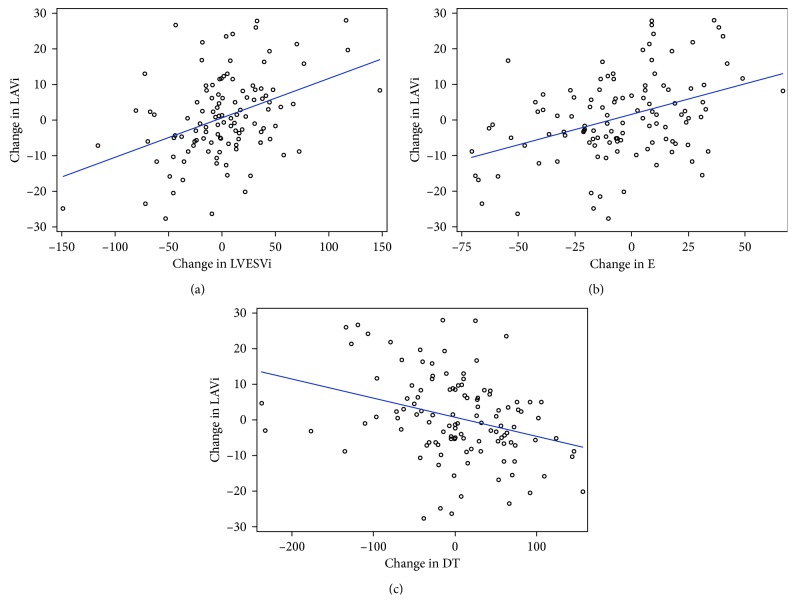
LAVi and diastolic function. Change in LVESVi (a) and E (b) are directly correlated with change in LAVi (*r*^2^=0.17 for LVESVi and *r*^2^=0.16 for E), whereas change in DT (c) is inversely correlated with change in LAVi (*r*^2^=0.10). LVESVi, indexed left ventricular end-systolic volume; E, early mitral inflow velocity; DT, deceleration time; LAVi, indexed left atrial volume.

To explore the factors that may affect subjects being in the normal or dilated LAVi groups, a multivariate logistic regression was conducted using a stepwise variable selection approach with *p* value of forward selection and backward selection both equal to 0.2. For the multivariate stepwise selection process, the significant level is usually larger than the traditional level of significance. As discussed by Bendel and Afifi [11] and Mickey and Greenland [12], more traditional significant levels such as 0.05 can fail in identifying variables known to be important. Choosing a larger level of significance helps to keep both type I and type II errors at a reasonable range. The relationship of LAVi and selected potential risk factors was also examined with scatter plots (Figure 3), and the R-squares for the corresponding linear regression were reported. The multivariate logistic model had the LAVi group as a dependent variable and all the investigated parameters as independent variables, including age, body mass index, race, baseline indexed left ventricular end-diastolic volume (LVEDVi), baseline indexed left ventricular end-systolic volume (LVESVi), creatinine, ACE-I use, nitrate use, LVEF, early and late mitral inflow velocities and their ratio, deceleration time, septal, mitral, and average mitral annular velocities, and the ratio of the mitral inflow velocity to the average mitral annular velocity.

To better understand the impact factors for LAVi changes, we applied a multivariate general linear model using the same stepwise variable selection technique, with *p* value of forward selection and backward selection both equal to 0.2 for the 6-month follow-up cohort and 0.05 for the 1-year follow-up subgroup. The dependent variable for this multivariate general linear model was the LAVi change between the two assessments, and the independent variables included age, race, baseline LVEDVi and LVESVi, creatinine, ACE-I use, nitrate use, LVEF, early and late mitral inflow velocities and their ratio, deceleration time, septal, mitral, and average mitral annular velocities, the ratio of the mitral inflow velocity to the average mitral annular velocity, and the changes in these variables between the two assessments. The final results were presented as variables selected into the final model, along with the variance of each of these variables explained in LAVi variance seen in this patient population. Both of the above analyses were done in the original cohort as well as a subgroup with at least 1 year between the baseline and follow-up TTE. Statistical analyses were performed using STATA 13.0 (College Park, Texas) and SAS 9.4 (SAS Institute Inc., Cary, NC, USA).

## 3. Results

### 3.1. Study Population

We found 816 patients in the normal LAVi group and 1094 in the dilated LAVi group. The majority of patients met at least one exclusion criterion (Figure 1); thus, we had 51 normal and 64 dilated LAVi subjects that were included for the area-length measurements. Based on the measured LAVi on both TTEs, the patients were further classified into persistently normal (*n*=26), persistently dilated (*n*=48), or change (increased, *n*=20; decreased, *n*=21) in LAVi. In the 115 patients included for analysis, the mean age was 58.4 ± 14.2 years and 32% were female. Demographic and baseline characteristics of persistently normal and persistently dilated subjects are summarized in Table 1. Mean LVEF at the time of the first TTE was 26.5 ± 7.8% and at the time of the second TTE was 26.4 ± 7.8%. 94 subjects were included in the subgroup with 1 year follow up.

### 3.2. Factors Associated with Normal LAVi

Several baseline characteristics were significantly different between the normal and dilated groups including age (52.0 ± 9.1 versus 64.9 ± 13.9 years, *p* < 0.01), body mass index (30.2 ± 7.1 versus 26.7 ± 5.4 kg/m^2^, *p*=0.02), N-terminal pro-brain natriuretic peptide (874.5 ± 1324.7 versus 9364.2 ± 11,296.3 mg/dL, *p*=0.02), creatinine (1.0 ± 0.3 versus 1.9 ± 1.9 mg/dL, *p*=0.04), and LVEF (29.9 ± 5.4% versus 24.1 ± 7.0%, *p* < 0.01; Table 1).

With the stepwise variable selection, the multivariate logistic regression model selected the following five risk factors, which independently predicted the probability of maintaining a normal LAVi over time: younger age (OR 1.18, CI 1.06–1.31, *p* < 0.01), white race (OR 7.43, CI 0.90–61.52, *p*=0.06), being on ACE-I therapy (OR 3.67, CI 0.54–25.17, *p*=0.19), decreased LVEDVi (OR 1.04, CI 0.99–1.08, *p*=0.11), and longer deceleration time (OR 1.02, CI 1.00–1.04, *p*=0.03). The model predicted an area under the curve of 0.95, *p* < 0.0001 (Table 2).

Subgroup analysis on subjects with one year between the initial and follow-up echo was performed. The multivariate logistic regression model selected the same set of clinical variables but a different diastolic parameter which independently predicted the probability of maintaining a normal LAVi over time: younger age (OR 1.22, CI 1.06–1.42, *p* < 0.01), white race (OR 5.62, CI 0.55–57.49, *p*=0.15), being on ACE-I therapy (OR 6.73, CI 0.66–68.34, *p*=0.11), and higher E-to-A ratio (OR 8.84, CI 1.05–74.29, *p*=0.05). The model predicted an area under the curve of 0.94, *p* < 0.0001 (Table 2).

### 3.3. Factors Associated with Change of LAVi

Although on average LAVi did not change appreciably over time (the mean difference in LAVi between time points 1 and 2 was 0.6 ± 11.8 ml/m^2^), there were many patients who experienced a significant increase or decrease in LAVi over the 2 time points. Using a multivariate model with stepwise variable selection, we found that changes in LVESVi (0.09 ± 0.02, *p* < 0.01, variance 15%), early mitral inflow velocity (0.12 ± 0.03, *p* < 0.01, variance 11%), and deceleration time (−0.04 ± 0.01, *p* < 0.01, variance 6%) between time points 2 and 1 were each significantly associated with change in LAVi (total variance accounted for was 32%; Table 3). Change in LVESVi and early mitral inflow velocity were directly associated with change in LAVi, and change in mitral annular deceleration time was indirectly associated with change in LAVi, as shown in Figure 3.

Subgroup analysis and the multivariate model with stepwise variable selection on patients with one year between the initial and follow-up TTE similarly showed that changes in early mitral inflow velocity (0.16 ± 0.04, *p* < 0.01, variance 13%) and LVESVi (0.09 ± 0.03, *p* < 0.01, variance 11%) were associated with a change in LAVi (total variance accounted for was 24%; Table 3).

## 4. Discussion

We examined a group of patients with reduced LVEF and normal LA size for clinical factors and echocardiographic parameters which distinguished them from their counterparts with severely dilated LA. Baseline factors including younger age, white race, being on ACE-I therapy, decreased LVEDVi, higher early-to-late mitral inflow velocity, and longer deceleration time were independently associated with having a normal LAVi. Longitudinally, an increase in LVESVi and early mitral inflow velocity as well as a decrease in deceleration time was associated with an increase in LAVi.

In this study, we found younger age to be an independent predictor of normal LA size in the setting of reduced LVEF. A number of studies have similarly demonstrated that LAVi increases with increasing age [9, 13–16]. Nikitin et al. measured phasic atrial volumes on healthy subjects with normal systolic function aged 22 to 89 years. They found that while LA reservoir and pump function are relatively preserved across all ages, LA conduit function (LA passive emptying fraction) is robust among the younger population [13]. LA passive emptying fraction is directly influenced by the pressure gradient across the mitral valve during the early diastole. Low LV end-diastolic pressure creates a large pressure gradient between the chambers and allows for a significant fraction of LA passive emptying during the early diastole. As LV end-diastolic pressures rise due to age-related LV wall stiffness, equalization of pressure across the 2 chambers ensues and the passive emptying fraction reduces [17]. In the absence of underlying cardiomyopathy, mild diastolic dysfunction develops with increasing age causing an increase in LV end-diastolic pressures and decrease in LA passive filling [18]. Younger age seems to be protective of normal diastolic filling and thus normal LA conduit function demonstrated by higher peak early mitral inflow velocities in younger people [13, 19]. Boyd et al. found this protective effect of age on atrial volume to persist until the eighth decade of life, implying that LA enlargement at any time point earlier likely reflects a pathological change apart from normal aging [15]. We have shown that even among patients with a cardiomyopathy such as moderate-to-severely depressed LVEF, younger age still remains a protective factor on the persistence of normal LA size.

In our study, patients with normal LA size were more likely to be white compared to other nonwhite races. There is little known about the relationship between the race and LA size. The Cardiovascular Health Study examined LAVi as a predictor of prevalent and incident heart failure and found that control subjects free of cardiovascular disease were less likely to be black and a higher percentage of black patients with prevalent heart failure had systolic heart failure compared to diastolic heart failure [20]. In a recent publication from the Multi-Ethnic Study of Atherosclerosis, Chinese Americans were found to have a smaller LAVi, while Hispanics had a larger LAVi as compared to whites [21]. We were not able to further investigate the interaction between different nonwhite races in our study due to a relatively small group of nonwhite patients. Larger research studies enrolling subjects of different races are needed to clarify the interaction between the race, LA size, and LV dysfunction.

The relationship of the LA size and diastolic function has been extensively studied in patients with normal LVEF [3, 22–24]. Tsang et al. found a strong positive linear relationship between LAVi and severity of diastolic dysfunction from abnormal to pseudonormal to restrictive [5]. Pritchett et al. showed that LAVi increases with increasing diastolic dysfunction as measured by early and late mitral inflow velocities, their ratio, and deceleration time with incremental increase in LAVi with worsening diastolic dysfunction [3]. The prognostic implication of increased LA size on mortality is enhanced after adjusting for clinical and echocardiographic parameters of systolic and diastolic function in people with cardiovascular disease such as acute myocardial infarction compared to the general population [25]. Little is known about the relationship between diastolic dysfunction and LA size in patients with concomitant systolic dysfunction. Rihal et al. analyzed Doppler TTE variables in patients with idiopathic dilated cardiomyopathy and found that, in addition to systolic blood pressure and LVEF, a restrictive filling pattern correlated with worse long-term survival in univariate analysis [26]. Additionally, Nishimura et al. noted that increasing LA pressure correlated with Doppler evidence of diastolic dysfunction in patients with systolic dysfunction defined as LVEF < 40% [27]. Similarly in our study, an increase in early mitral inflow velocity and a decrease in deceleration time over a period of 6 months or more were associated with an increase in LAVi. Subgroup analysis showed that a higher early-to-late mitral inflow velocity at baseline was associated with maintaining a normal LAVi at least one year later on follow-up TTE. This along with the association of longer deceleration time and normal LAVi supports the notion that less severe restrictive filling patterns or less severe diastolic dysfunction at baseline is associated with long-term left atrial integrity. Our findings support the concept that diastolic dysfunction is associated with severity of LA enlargement, and additionally, perhaps incremental changes in diastolic function in the presence of systolic dysfunction have a more profound impact on LAVi. This in turn has a significant impact on using parameters including E-to-A ratio, DT, and LAVi together in risk-stratifying patients by the presence and/or severity of diastolic dysfunction.

In the present study, smaller LVEDVi correlated with having a normal LAVi, consistent with our physiological understanding of pressure volume loops. Zile et al. studied patients with clinical heart failure with a normal LVEF and plotted pressure volume loops that showed that, in patients with a normal LVEF, a small increase in LVEDVi results in an exponential increase in LV end-diastolic pressure, indicating low compliance. These patients are said to have diastolic dysfunction arising from a stiff ventricle [28]. Patients with dilated cardiomyopathy and systolic dysfunction according to Glantz and Parmley have Frank–Starling curves that are shifted rightward and flattened with increased compliance [29]. However, the principle still holds true that increasing LVEDVi will increase end-diastolic pressure over time. Patients with systolic dysfunction but normal LAVi must have some mechanism of preserving compliance such that LV filling pressures do not rise sufficiently to cause long-term atrial remodeling and enlargement. Younger age and smaller LVEDVi might partially explain the preservation of diastolic compliance. In our study, indicators of worsening systolic function (increased LVESVi) and indicators of worsening diastolic function (elevated early mitral inflow velocity) were associated with an increase in LAVi overtime. Taken together, this signifies that left ventricular systolic and diastolic functions are closely interrelated and subsequently affect the left atrial structure and function.

Our study is limited by the small sample size and the retrospective nature of the study. The small sample size of the normal LAVi group in the setting of LV systolic dysfunction might be inherent in the patient population that we were attempting to study. Additionally, we used echocardiographic parameters to estimate diastolic function due to limited availability of cardiac catheterization data, and isovolumetric relaxation time was not included in the parameters for assessment of diastolic function.

## 5. Conclusion

In a carefully assessed group of patients with moderate-to-severely reduced LVEF, younger age, white race, being on ACE-I therapy, smaller LVEDVi, and longer deceleration time were associated with maintaining a normal LAVi over a period of at least 6 months. Over a longer period of a year, in addition to the clinical parameters, better systolic performance as indicated by smaller LVESVi and better diastolic performance were associated with decreased LAVi.

## Figures and Tables

**Table 1:** Baseline demographics and clinical characteristics.

Variable	Normal LAVi (*n*=26)	Dilated LAVi (*n*=48)	*p* value
Age (years) (mean ± SD)	52.0 ± 9.1	64.9 ± 13.9	<0.01
Medications, *n* (%)			
ACE-I	17 (65)	21 (44)	0.08
ARB	5 (19)	16 (33)	0.20
Hydralazine	0	8 (17)	—
Oral nitrates	1 (4)	10 (21)	0.08
AA	7 (27)	11 (23)	0.70
BB	26 (100)	43 (90)	—
CCB	1 (4)	4 (8)	0.47
Digoxin	3 (12)	9 (19)	0.43
Statin	19 (73)	29 (60)	0.28
Diuretic	18 (69)	40 (83)	0.17
Anticoagulation	7 (27)	13 (27)	0.99
Antiplatelet	21 (81)	36 (75)	0.57
Female, *n* (%)	8 (31)	14 (29)	0.89
BMI (kg/m^2^) (mean ± SD)	30.2 ± 7.1	26.7 ± 5.4	0.02
White race, *n* (%)	18 (69)	18 (38)	0.01
Previous myocardial infarction, *n* (%)	14 (54)	27 (56)	0.84
Coronary artery disease, *n* (%)	14 (54)	28 (58)	0.71
Ischemic cardiomyopathy, *n* (%)	12 (46)	26 (54)	0.55
CABG, *n* (%)	5 (19)	10 (21)	0.87
Hypertension, *n* (%)	13 (50)	31 (65)	0.23
Hyperlipidemia, *n* (%)	18 (69)	31 (65)	0.69
Diabetes, *n* (%)	14 (54)	16 (33)	0.09
Stroke/TIA, *n* (%)	3 (12)	9 (19)	0.43
Obstructive sleep apnea, *n* (%)	6 (23)	4 (8)	0.09
Smoking history, *n* (%)	17 (65)	29 (60)	0.84
NT-proBNP (mg/dL) (mean ± SD)	874.5 ± 1324.7	9364.2 ± 11,296.3	0.02
Creatinine (mg/dL) (mean ± SD)	1.0 ± 0.3	1.9 ± 1.9	0.04
Hemodialysis, *n* (%)	0	2 (4)	—
PCWP (mmH_2_O) (mean ± SD)	14.1 ± 6.7	22 ± 8.2	0.07
LVEF (%) (mean ± SD)	29.9 ± 5.4	24.1 ± 7.0	<0.01

ACE-I, angiotensin-converting enzyme inhibitor; ARB, angiotensin receptor blocker; AA, aldosterone antagonist; BB, beta-blocker; CCB, calcium channel blocker; BMI, body mass index; CABG, coronary artery bypass grafting; TIA, transient ischemic attack; NT-proBNP, N-terminal pro-brain natriuretic peptide; PCWP, pulmonary capillary wedge pressure; LVEF, left ventricular ejection fraction.

**Table 2:** Factors associated with a normal LAVi.

Variable	6-month interval			1-year interval		
	OR	95% CI	*p* value	OR	95% CI	*p* value
Younger age	1.18	1.06–1.31	<0.01	1.22	1.06–1.42	<0.01
White race	7.43	0.90–61.52	0.06	5.62	0.55–57.49	0.15
ACE-I therapy	3.67	0.54–25.17	0.19	6.73	0.66–68.34	0.11
Decreased LVEDVi	1.04	0.99–1.08	0.11			
Longer DT	1.02	1.00–1.04	0.03			
E-to-A ratio				8.84	1.05–74.29	0.05

The model suggested that the following risk factors are associated with an increased probability of having a normal LAVi with 6-month interval and 1-year interval between baseline and follow-up TTEs: younger age, white race, being on ACE-I therapy, smaller LVEDVi, longer DT, and higher E-to-A ratio. The model predicted an area under the curve of 0.95 (*p* < 0.0001) for the 6-month interval and 0.94 (*p* < 0.0001) for the 1-year interval. ACE-I, angiotensin-converting enzyme inhibitor; LVEDVi, indexed left ventricular end-diastolic volume; DT, deceleration time; E-to-A ratio, early-to-late mitral inflow velocity ratio; LAVi, indexed left atrial volume.

**Table 3:** Factors associated with change in LAVi.

Variable	6-month interval			1-year interval		
	Estimate ± standard error	*p* value	Variance	Estimate ± standard error	*p* value	Variance
LVESVi	0.09 ± 0.02	<0.01	15%	0.09 ± 0.03	<0.01	11%
E	0.12 ± 0.03	<0.01	11%	0.16 ± 0.04	<0.01	13%
DT	−0.04 ± 0.01	<0.01	6%			

Change in LVESVi and E were directly associated with change in LAVi at 6-month and 1-year follow-up, and DT was inversely associated with change in LAVi at 6-month follow-up. Total variance accounted for by the above factors was 32% at 6-month follow-up and 24% at 1-year follow-up. LVESVi, indexed left ventricular end-systolic volume; E, early mitral inflow velocity; DT, deceleration time; LAVi, indexed left atrial volume.
